# Hepatotoxicity-Related Adverse Effects of Proton Pump Inhibitors: A Cross-Sectional Study of Signal Mining and Analysis of the FDA Adverse Event Report System Database

**DOI:** 10.3389/fmed.2021.648164

**Published:** 2021-11-15

**Authors:** Yifan Zeng, Ying Dai, Ziye Zhou, Xuben Yu, Dawei Shi

**Affiliations:** ^1^Computer Technology and Information Centre, First Affiliated Hospital of Wenzhou Medical University, Wenzhou, China; ^2^Department of Pharmacy, First Affiliated Hospital of Wenzhou Medical University, Wenzhou, China

**Keywords:** proton pump inhibitors, hepatotoxicity, cholestasis, coma hepatic, hepatitis fulminant

## Abstract

**Background and Objectives:** Mounting evidence demonstrates that proton pump inhibitors (PPIs) are associated with a number of adverse effects. However, the literatures about hepatotoxicity-related adverse effects (HRAEs) of PPIs are mostly case reports and a few clinical studies.

**Methods:** We evaluated the association between PPIs and HAREs using the reporting odd ratio (ROR) for mining the adverse event report signals in the FDA Adverse Event Reporting System (FAERS) database.

**Results:** There were 23,825 reports of PPIs as primary suspect drug or second suspect drug, of which 3,253 reports were HRAEs. The top five HRAE signals caused by PPIs were hepatitis cholestatic, cholestasis, fulminant hepatitis, subacute hepatic failure, and acute hepatitis. We also summarized the signals of the HRAEs caused by each PPI. The simultaneous signals were cholestasis and hepatitis cholestatic. For the cholestasis signal, esomeprazole showed an ROR of 21.556 (95% CI 17.592–26.413); pantoprazole showed the highest ROR of 22.611 (95% CI 17.794–28.733) in the hepatic cholestatic signal; lansoprazole was the only PPI with expression in the coma hepatic signal, with an ROR of 10.424 (95% CI 3.340–32.532). By analyzing the reports of pantoprazole-induced hepatic encephalopathy, we found that patients aged over 65 years and males reported the highest rate. And from the combination of drugs and indications of drugs, no significant results were obtained.

**Conclusions:** The RORs of signals of “cholestasis” were generally higher than those of “hepatocellular injury.” And the signals about “cholestasis” in HRAE caused by PPIs are more reported.

## Introduction

Proton pump inhibitors (PPIs) are a class of medications that work to decrease gastric acid and are FDA-approved for the treatment of a variety of acid-related conditions, including duodenal ulcers, gastric ulcers, erosive esophagitis, gastroesophageal reflux disorder, Helicobacter pylori eradication, and pathological hypersecretory conditions (1). Since the introduction of omeprazole (OME) in 1989, PPIs have demonstrated consistent patient tolerance and excellent safety compared with prior agents (2). Recently, mounting evidence has demonstrated that PPIs are related to a number of adverse effects, including acute and chronic kidney disease, hypomagnesemia, *Clostridium difficile* infection, pneumonia, and osteoporotic fractures, and so on (3). However, the literature about hepatotoxicity-related adverse effects (HRAEs) of PPIs are mostly case reports and a few large clinical studies (4–6). We also found in VigiAccess of the Uppsala Monitoring Center that HRAEs caused by PPIs are not common (7). Taking Omeprazole as an example, among the 108,954 cases reported, only 2,156 reports from the hepatobiliary system were reported, accounting for only 2.0% of the reports. Therefore, physicians tend to ignore the HRAEs caused by PPIs in clinical practice.

The FDA Adverse Event Reporting System (FAERS) includes several million spontaneous reports of drug-associated adverse events and is used to evaluate drug safety profiles (8, 9). It is one of the primary tools used for post-marketing surveillance and pharmacovigilance because it is the largest, most well-known database worldwide, and it reflects the realities of clinical practice.

In our study, adverse event reports submitted to the FDA were reviewed to assess the adverse event profiles of 7 PPIs: omeprazole, esomeprazole, lansoprazole, dexlansoprazole, rabeprazole, pantoprazole, and ilaprazole. Data mining algorithms were used for the quantitative detection of signals, in which a signal represents an association between a drug and an adverse event (10, 11). The adverse events related to hepatotoxicity were analyzed. To our knowledge, our study was the first to evaluate the association between PPIs and HRAEs using the reporting odds ratio (ROR) for mining the adverse event report signals in the FAERS database (12).

## Methods

### Data Source

The study was designed as a retrospective study, and adverse drug event quarterly report files from Jan 2013 to Dec 2019 in the FAERS database were downloaded from the FDA website (13). These files contain reports on adverse drug events submitted by physicians, pharmacists, other healthcare professionals, manufacturers, and consumers from the U.S. and other countries. We built a database that integrated the quarterly report files using Oracle Database 11g software (Oracle, USA). The drugs selected for this investigation were omeprazole, esomeprazole, lansoprazole, dexlansoprazole, rabeprazole, pantoprazole, and ilaprazole. Before analyzing the data, a text-mining approach was utilized that stated the drugs in terms of their generic names and brand names. We obtained the brand names of each PPI by querying the DrugBank database online. The DrugBank database is a comprehensive, freely accessible, online database containing information on drugs and drug targets (14). Then, we set the target drug as the primary suspect drug (PS) or second suspect drug (SS). We followed the FDA's recommendation to adopt the most recent and unique case number to identify duplicate reports of the same patient with different reporting sources and excluded these from the analysis. Then, a second process was performed using a record-linkage strategy, which groups records overlapping in three key fields: date the FDA received the first version of the case, age and sex of the patient and reporter country. Records with three overlaps were also considered duplicates.

### Definition of Hepatotoxicity Events

This study relied on the definitions provided by MedDRA version 23.0 (15). To evaluate the effect of PPI treatment on HRAEs, different preferred terms (PTs) were identified with the Standardized MedDRA Query (SMQ) for “livery injury” and “acute hepatic failure” and the System Organ Class (SOC) for hepatobiliary disorder, and only reports that met both criteria were extracted. The number of selected PTs for “livery injury” and “acute hepatic failure” was 40 (see details in Supplementary Table S1).

Then, according to the main clinical features of adverse events, the selected PTs were divided into three types: “hepatocellular injury,” “cholestasis,” and “liver failure.”

### Analysis

All reported adverse events of interest were defined as “HRAE cases,” and all reported other adverse events were defined as “no-HRAE cases.” To compare one of the PPI groups with the no-PPI group, we calculated the RORs as (a: c)/ (b: d). RORs are expressed as point estimates with 95% confidence intervals (CIs). For signal detection, general qualitative judgments are viable, and whether a signal is detected or not depends on whether the signal indices exceed predefined thresholds: ROR values >1 and the number of total reports ≥3 indicate potential exposure-event signals (16). Meanwhile, we defined the exposure-event signal of ROR value ≥5 as the “strong signal.”

This study analyzed the occurrences of HRAEs caused by PPIs and mainly included four aspects. First, the RORs of HRAEs caused by PPIs vs. non-PPI drugs were calculated. Second, we screened the “strong signal” adverse events in PPI-induced HRAEs. Third, the “strong signal” adverse events in HRAEs co-owned by different PPIs were screened. Fourth, the RORs of “strong signal” adverse events in HRAEs co-owned by different PPIs were calculated.

We also analyzed the reports of the adverse events related to hepatic encephalopathy, which are more clinically concerning. We mainly mined the basic characteristics, clinical diagnosis and combined medication information of the patients in these reports.

Statistical significance was verified using chi-square tests. Data processing and analysis were conducted using SPSS version 25.0. Differences with *P*-values of <0.05 were considered statistically significant.

## Results

After the exclusion of duplicates following the FDA recommendation, 8,221,278 reports in the FAERS database were of use. We used the generic name and brand name of the target drug to search the database, and the details are shown in Supplementary Table S2. There were 23,825 reports of PPIs considered as PS and SS, of which 3,253 reports were HRAEs. Because the adverse event reports of dexlansoprazole and ilaprazole were not found in the database, we omitted them in the final analysis. Thirty-one HRAEs were defined as exposure-event signals of the 40 caused by PPIs. The top five HRAE signals caused by PPIs were hepatitis cholestatic, cholestasis, fulminant hepatitis, subacute hepatic failure, and acute hepatitis, and the RORs were 13.777 (95% CI 11.679–16.252), 12.939 (95% CI 11.795–14.193), 8.315 (95% CI 6.254–11.055), 7.301 (95% CI 2.292–23.259), and 6.823 (95% CI 5.560–8.373), respectively. The number of cases and RORs of HRAEs are detailed in Table 1.

**Table 1:** The number of cases and RORs of HRAE associated with all PPIs.

**PT**	**a**	**ROR (95% two-sided CI)**	***P***
Acute hepatic failure	103	3.262 (2.683–3.966)	<0.001
Alanine aminotransferase abnormal^*^	3	0.682 (0.219–2.119)	0.808
Ammonia increased^*^	3	0.213 (0.069–0.661)	0.001
Alanine aminotransferase increased	249	2.052 (1.810–2.325)	<0.001
Aspartate aminotransferase increased	171	1.687 (1.450–1.962)	<0.001
Bilirubin conjugated increased	18	3.579 (2.243–5.712)	<0.001
Blood bilirubin increased	110	1.783 (1.477–2.152)	<0.001
Cholestasis	491	12.939 (11.795–14.193)	<0.001
Coma hepatic	5	2.826 (1.166–6.845)	0.035
Hepatic encephalopathy	66	2.669 (2.092–3.405)	<0.001
Hepatic enzyme abnormal^*^	13	1.036 (0.600–1.787)	0.887
Hepatic enzyme increased	237	1.867 (1.642–2.123)	<0.001
Hepatic failure	130	1.854 (1.559–2.205)	<0.001
Hepatic function abnormal	187	2.416 (2.090–2.792)	<0.001
Hepatic necrosis	33	4.957 (3.504–7.012)	<0.001
Hepatitis	236	4.585 (4.027–5.220)	<0.001
Hepatitis acute	96	6.823 (5.560–8.373)	<0.001
Hepatitis cholestatic	154	13.777 (11.679–16.252)	<0.001
Hepatitis fulminant	50	8.315 (6.254–11.055)	<0.001
Hepatitis toxic	16	2.775 (1.693–4.551)	<0.001
Hepatotoxicity	100	2.233 (1.833–2.721)	<0.001
Hyperammonaemia	22	1.828 (1.201–2.784)	0.009
Hyperbilirubinemia	42	1.699 (1.253–2.303)	0.001
Jaundice	218	3.283 (2.870–3.755)	<0.001
Jaundice cholestatic	31	3.926 (2.748–5.608)	<0.001
Liver function test abnormal	160	2.251 (1.926–2.632)	<0.001
Liver injury	122	3.337 (2.788–3.993)	<0.001
Liver transplant^*^	3	0.332 (0.107–1.032)	0.043
Subacute hepatic failure	3	7.301 (2.292–23.259)	0.009
Transaminases abnormal^*^	3	1.748 (0.560–5.455)	0.249
Transaminases increased	170	3.074 (2.640–3.578)	<0.001

*HRAE, Hepatotoxicity-Related Adverse Effect; PPIs, Proton Pump Inhibitors; PT, Preferred Terms*;

* *not defined signal of HRAE*.

**Figure 1:**
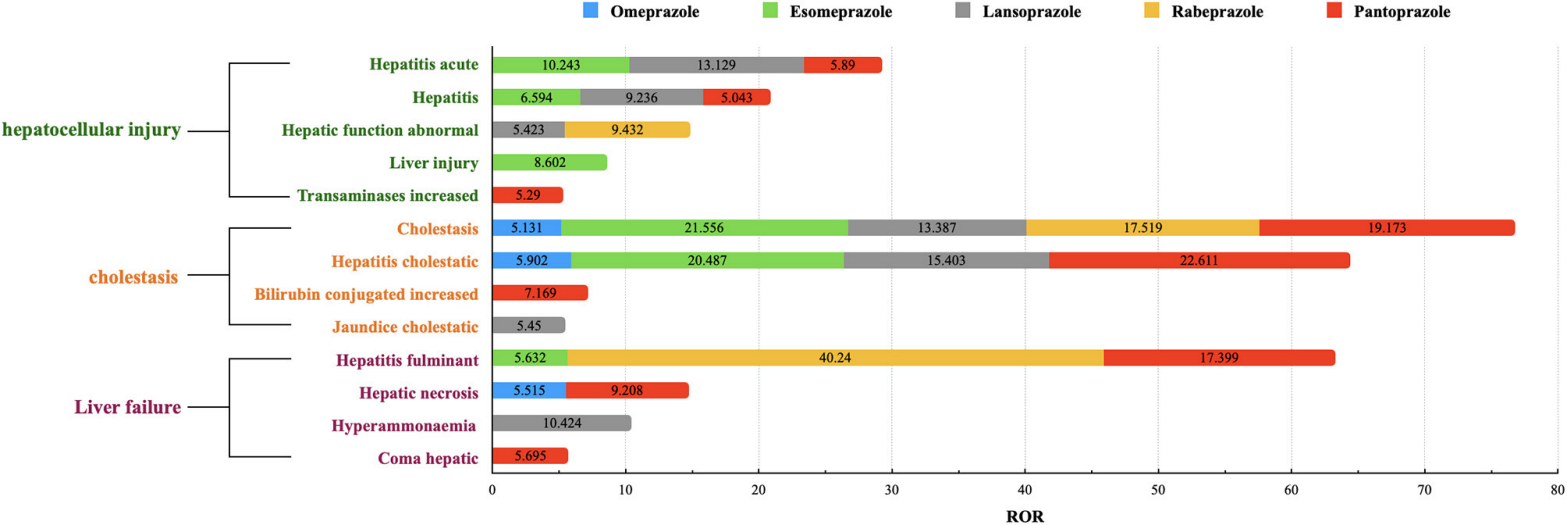
The signals of RORs ≥5 in each PPI. The ROR value shown in this figure is a ROR value of a HRAE signal caused by a single PPI. We extracted signals with ROR ≥ 5 for each PPI, and integrated the RORs of the same signal to get this figure.

We also summarized the signals of the HRAEs caused by each PPI (see details in Supplementary Tables S3–S7) and extracted 13 different signals for each PPI with ROR ≥ 5 (see details in Figure 1). Among them, the signals of “hepatocellular injury” included acute hepatitis, hepatitis, liver injury, hepatic function abnormal and transaminases increased. The signals of “cholestasis” included cholestatic, hepatitis cholestatic, bilirubin conjugated increased and jaundice cholestatic. “Liver failure” included signals such as hepatitis fulminant, hepatic necrosis, coma hepatic, and hyperammonemia.

We found that the RORs of signals of “cholestasis” were generally higher than those of “hepatocellular injury.” The signals of “liver failure” were mainly distributed in two or three PPIs. The simultaneous signals of more than four kinds of PPIs were cholestasis and hepatitis cholestatic. Pantoprazole had the most signal expression among the 13 signals, followed by lansoprazole. For the cholestasis signal, esomeprazole showed an ROR of 21.556 (95% CI 17.592–26.413), which was higher than that of the other PPIs; Pantoprazole showed the highest ROR of 22.611 (95% CI 17.794–28.733) in the hepatic cholestatic signal; Lansoprazole was the only PPI with expression in the coma hepatic signal, with an ROR of 10.424 (95% CI 3.340–32.532); Rabeprazole and pantoprazole showed expression the hepatitis fulminant signal, with RORs of 40.240 (95% CI 20.032–80.834) and 17.399 (95% CI 12.017–25.191), respectively.

As shown in Figure 1, we found that the RORs of pantoprazole were higher in the signals of adverse events related to hepatic encephalopathy, therefore, pantoprazole was selected as the representative drug for subsequent information analysis. In the 28 reports of pantoprazole-related hepatitis encephalopathy, the proportion of men was higher than that of women, accounting for ~68%. The proportion of patients aged ≥60 years was highest and was close to two-thirds. Reports of pantoprazole combined with more than three drugs were most frequent, accounting for ~71%. Antibiotics, antilipemic agents and contrast agents were commonly used drugs in combination with pantoprazole. The common clinical diagnosis when pantoprazole was used was skin infection (see details in Tables 2, 3 and Supplementary Table S8).

**Table 2:** The number of annual reports and clinical characteristics in Pantoprazole reports about hepatic encephalopathy.

**Year**	**Number of reports (*n*)**
2013	0
2014	0
2015	3
2016	0
2017	10
2018	14
2019	1
**Clinical characteristics**	**% (** ***n*** **)**
**Sex**	
Male	19 (67.86%)
Female	6 (21.43%)
Unknown	3 (10.71%)
**Age (y, year)**	
<18	0 (0.00%)
≥18 and <35	2 (7.14%)
≥35 and <60	3 (10.71%)
≥60	19 (67.86%)
Unknown	4 (14.29%)

**Table 3:** Frequency of concomitant medication occurrences in Pantoprazole reports about hepatic encephalopathy.

**Concomitant medication**	**Frequency of occurrence *n* (%)**
Single drug	0
Combined 1 drug	0
Combined 2 drugs	5 (17.86%)
Combined 3 drugs	3 (10.71%)
Combined more than 3 drugs	20 (71.43%)
Total	28

## Discussion

Abnormal liver enzymes (alanine aminotransferase, aspartate aminotransferase, alkaline phosphatase, etc.) are generally regarded as common adverse events in clinical studies of PPIs or in the labels for the drug (17–20). In our study, although aspartate aminotransferase increased, liver function test abnormal and transaminases increased were defined as signaling adverse events, their ROR values were significantly different from those of cholestasis-associated signals (see details in Table 2). We think that there are several reasons for this result. First, mild liver enzyme elevation usually has fewer clinical manifestations and can only be diagnosed by relevant biochemical detection. Therefore, this situation results in a low report rate of liver enzyme abnormalities. Second, some reports tend to describe clinical manifestations, such as hepatitis and liver injury, in which abnormal liver enzymes may coexist but were not reported.

Although abnormal liver enzymes may be underreported, there is no doubt that reports related to cholestasis have a stronger signal. In this study, we found that cholestatic signals were more likely to be detected both in all PPI HRAE reports and in individual PPI HRAE reports, which is not consistent with the information we obtained from the literature. Moreover, other signals related to “cholestasis” were reported at high rates. It may be that there were more obvious clinical manifestations in these patients or more severe symptoms, so cholestasis was therefore more widely reported.

We also screened the signals of each PPI, and the “strong signal” expression of each PPI was similar to that of all PPIs. Moreover, we also found that for the co-owned signals of the PPIs, only cholestasis was identified, suggesting that there was a significant difference in the “strong signal” between PPIs. Except for omeprazole, the other PPIs had high ROR values. This may be related to the fact that omeprazole was the first PPI to be used, and then influenced by Weber effect (21), the reports would decline year by year. Although it is not always observed (22). We also found that the RORs of the “cholestasis” signals were generally higher than those of “hepatocellular injury.” This may be due to that “cholestasis” is more likely to cause doctors' influence than “hepatocellular injury.” On the other hand, it could also be considered as a ripple effect (23).

In the single signal analysis, a “strong signal” of hepatitis fulminant was expressed in rabeprazole. Among all the PPIs, due to its late market time and small market scope, rabeprazole had the lowest number of reports, which were 1,797, and the number of HRAE reports was 133. Since the ROR of hepatitis fulminant was very high (ROR = 40.240, 95% CI 20.032–80.834), the authors believed that this could possibly be a false positive result because the data volume was small, which means that the frequency method could easily exaggerate the results and obtain many false positive results (16). The same reason was applied to the signal of coma hepatic. The ROR value of lansoprazole was high (ROR = 10.424, 95% CI 3.340–32.532), but only 3 cases were reported.

**Figure 2:**
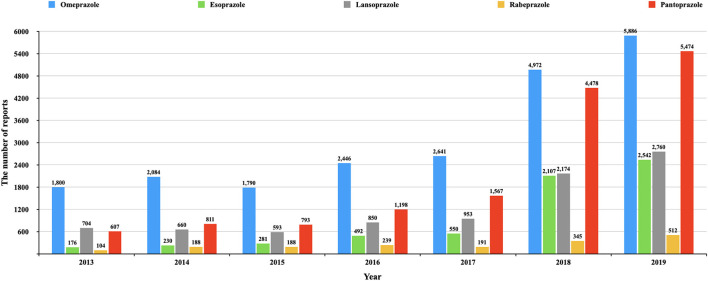
The number of reports of each PPI in different year. This figure shows the number of reports of each PPI in different year (from the first quarter of 2013 to the fourth quarter of 2019).

Omeprazole had the earliest clinical application and the most retrieved reports (22,117), but the number of reports of HRAEs (*n* = 913) was lower than that of pantoprazole (*n* = 1,134) (see details in Figure 2). The reported rates of adverse events in cholestasis and liver failure with pantoprazole were significantly higher than those with omeprazole. The signals of HARE in pantoprazole were the most common of all PPIs. This phenomenon also needs to be considered.

In recent years, more studies have revealed the relationship between hepatic encephalopathy and PPIs (24–26). In the above studies, the conclusions did not suggest which PPI had a higher risk of developing hepatic encephalopathy. Among all the signals about this adverse event, whether it was coma hepatic, hyperammonemia, or hepatic encephalopathy, there was also a higher reporting rate in a certain PPI. Among the PPIs with a high reporting rate in these signals, we selected pantoprazole as the representative drug to analyze the characteristics of the reports of hepatic encephalopathy. We found that most reports occurred in the past 3 years, consistent with the time when physicians were aware of such adverse events. In terms of the clinical characteristics of the patients, males aged over 65 years had the highest proportion in the reports (see details in Table 3). In a recent meta-analysis on the use of Proton pump inhibitor therapy and hepatic encephalopathy risk in cirrhotic patients, similar results were obtained, the proportion of male patients was higher than that of women, and most of them were middle-aged and elderly patients (27). This may be related to the epidemiological characteristics of patients with liver disease. Generally, patients with liver disease have a higher incidence of hepatic encephalopathy (28), and the proportion of male patients with liver disease was often higher than that of female patients (29, 30). However, in patients without liver diseases, the reports of hepatic encephalopathy caused by PPIs are rare (31), it is difficult to determine the gender differences. Furthermore, due to the limitations of the study (32), the reported rate of adverse events cannot be equated to the actual incidence of adverse events. Therefore, even though the proportion of male is higher in the report, further clinical studies are also needed to further clarify the relationship between gender and hepatic encephalopathy caused by PPIs. In addition, another clinical study found that the use of different PPI, liver cirrhosis patients with a slightly different risk of hepatic encephalopathy, in which the risk of pantoprazole was higher, and OR value was 2.05 (25).

We also tried to explore the clinical diagnosis of patients through the description of indications in the reports, but unfortunately, due to the defects of the reports themselves, many indications for drug use were not accurately described. The indications for some of the drugs that were obtained did not accurately reflect the extent to which underlying diseases contribute to adverse events (see details in Supplementary Table S8).

We described the situation of the combined use of drugs in the reports and found that 71.43% of the reports, more than three drugs were used at the same time (see details in Table 3). We also extracted information on drugs with a high frequency of combined use, which were mainly antibiotics, but did not find a clear causal relationship with the occurrence of adverse events (see details in Supplementary Table S8). Therefore, the information collected in the reports was similar to the results of clinical studies.

From a statistical point of view, adverse event signal strength can indicate the magnitude of the correlation between drugs and events. Therefore, it is currently believed that signal detection can preliminarily describe the possible relationship between drugs and adverse events and aid in further evaluation and research. At present, the only method that can be used for adverse event signal detection is the disproportionality method, including the proportional reporting ratio (PRR) method, ROR method, Bayesian method, etc. The ROR method was chosen because it is more sensitive and accurate than other methods (33). Considering that the main purpose of our study was to detect the signals of HRAE of PPIs, we did not continue to verify the results of the other methods in our study. This is also a limitation of the statistical results of the study.

This study has limitations as well. First, while the database has the merit of providing an early real-world perspective, the quality of the diagnosis of adverse events likely widely varies, and our study captures a short time frame early in the medication market life. And the study was a retrospective study, which only interpreted the reported data from the first quarter of 2013 to the fourth quarter of 2019. As a result, based on the data obtained from this period, the statistical results may have some limitations. Second, there is limited information in FAERS regarding a wide range of patient health characteristics, thus limiting the ability to control confounding effects (34). For example the association of a drug with an adverse event might be explained by those of other drugs which are often co-administered. For another example, PPIs are metabolized by CYP2C19; and so, the hepatotoxicity-related adverse effects could be affected by CYP2C19 genotype. Indeedly, the drug-drug interaction is subjected to CYP2C19 genotype (35). Of course, FAERS database is not included genotyping information. Third, since the reports of adverse events in FAERS are voluntary, it is difficult to complete the quality evaluation of the reports. Sometimes, the basic diseases of patients will also have adverse manifestations of liver and gallbladder (36, 37). Therefore, we retrieved the reported data of major adverse events with high ROR value of pantoprazole, and sorted out the indications of the top 10 of each HRAE to judge the patient's disease background. Unfortunately, we found that the main indication in the report was “PRODUCT USED FOR UNKNOWN INDICATION,” so it is difficult to distinguish whether the occurrence of adverse events is related to the underlying disease (see details in Supplementary Table S9). Fourth, adverse events are underreported in spontaneous reporting systems in general (38, 39). The rate of reporting can vary with the particular adverse event (40), but averages just 6% (38). Even though the reporting rate has dramatically improved, the FAERS database is still not appropriate for estimating incidence rates, due to the absence of a denominator (32). After considering causality restraints of the current analysis, it is recommended that robust epidemiological studies should be conducted to further validate the hypothesis to draw conclusions that contribute to clinical practice.

## Conclusion

This study found that the “strong signal” of HRAEs in PPIs were cholestasis and hepatitis cholestatic, whether in all the PPI HRAE reports or within a single species of PPI HRAE reports. Rabeprazole and pantoprazole were more likely to cause hepatitis fulminant than the other PPIs. By analyzing the reports of pantoprazole-induced hepatic encephalopathy, we found that patients aged over 65 years and males reported the highest rate, which was similar to the results of known clinical studies. However, from the combination of drugs and indications of drugs, no positive and significant results were obtained. Owing to the limitations of the study, further studies are needed to identify the effects of other combinations of drugs on PPI-induced HRAEs.

## Data Availability Statement

Publicly available datasets were analyzed in this study. This data can be found at: https://fis.fda.gov/extensions/FPD-QDE-FAERS/FPD-QDE-FAERS.html.

## Author Contributions

YZ and YD contributed to the data analysis and overall construction of the paper. XY contributed to the management of adverse events. ZZ peer reviewed the paper internally. DS conceived the paper and had overall oversight of the data analysis and paper construction. All authors contributed to the article and approved the submitted version.

## Conflict of Interest

The authors declare that the research was conducted in the absence of any commercial or financial relationships that could be construed as a potential conflict of interest.

## Publisher's Note

All claims expressed in this article are solely those of the authors and do not necessarily represent those of their affiliated organizations, or those of the publisher, the editors and the reviewers. Any product that may be evaluated in this article, or claim that may be made by its manufacturer, is not guaranteed or endorsed by the publisher.
