# Supplementary material for: Hepatotoxicity-Related Adverse Effects of Proton Pump Inhibitors: A Cross-Sectional Study of Signal Mining and Analysis of the FDA Adverse Event Report System Database
Source: Front Med (Lausanne). 2021 Nov 15;8:648164. doi: 10.3389/fmed.2021.648164 (PMC8636138; doi:10.3389/fmed.2021.648164)
Supplement: Supplementary file 1 [file Data_Sheet_1.docx]

Supplementary Materials

**Table S1: Preferred Terms of HRAE limited by Hepatobiliary disorder**

**Table S2: Generic and brand names of proton pump inhibitors**

**Table S3: The number and RORs of HARE reports of Omeprazole in different PT**

**Table S4: The number and RORs of HARE reports of Esomeprazole in different PT**

**Table S5: The number and RORs of HARE reports of Lansoprazole in different PT**

**Table S6: The number and RORs of HARE reports of Rabeprazole in different PT**

**Table S7: The number and RORs of HARE reports of Pantoprazole in different PT**

**Table S8: Ranking of indications and concomitant medications in Pantoprazole reports about hepatic encephalopathy**

**Table S9: Ranking of indications (Top 10) in Pantoprazole reports about major PT**

**Table S1: Preferred Terms of HRAE limited by Hepatobiliary disorder**

| **Preferred Terms: (liver injury and acute hepatic failure)** | |
| --- | --- |
| Acute hepatic failure | Hepatitis |
| Alanine aminotransferase abnormal | Hepatitis acute |
| Ammonia increased | Hepatitis cholestatic |
| Alanine aminotransferase increased | Hepatitis fulminant |
| Aspartate aminotransferase abnormal | Hepatitis toxic |
| Aspartate aminotransferase increased | Hepatocellular damage |
| Bilirubin conjugated increased | Hepatotoxicity |
| Bilirubin urine | Hyperammonaemia |
| Blood bilirubin abnormal | Hyperbilirubinaemia |
| Blood bilirubin increased | Jaundice |
| Blood bilirubin unconjugated increased | Jaundice cholestatic |
| Cholestasis | Jaundice hepatocellular |
| Coma hepatic | Liver function test abnormal |
| Cytolytic hepatitis | Liver injury |
| Hepatic encephalopathy | Liver transplant |
| Hepatic enzyme abnormal | Mixed hepatocellular-cholestatic injury |
| Hepatic enzyme increased | Subacute hepatic failure |
| Hepatic failure | Transaminases abnormal |
| Hepatic function abnormal | Transaminases increased |
| Hepatic necrosis | Urine bilirubin increased |

HRAE, Hepatotoxicity-Related Adverse Effect

**Table S2: Generic and brand names of proton pump inhibitors**

| **Generic name** | **Brand name** |
| --- | --- |
| omeprazole | Antra / Audazol / Belmazol / Ceprandal / Danlox / Desec / Elgam / Emeproton / Gasec / Gastrimut / Gastroloc / Indurgan / Inhibitron / Logastric / Losec / Mepral / Mopral / Olexin / Omapren / Omepral / Omeprazon / Omeprol / Omezol / Omisec / Omizac / Ortanol / Parizac / Prazidec / Prazolit / Procelac / Ramezol / Regulacid / Sanamidol / Ulceral / Ulcesep / Ultop / Zepral |
| esomeprazole | Alenia (Delta) / Awa-Block (Usawa) / Axagon (Simesa) / Cor (Prater) / Cronopep (Biotoscana) / Emanera (Krka) / Emep (Aristopharma) / Emozul (HYGIA) / ES-OD (Piramal Healthcare) / Esmep (HYGIA) / Eso (Asiatic Lab) / Esofag (Micro Labs) / Esolok (Ibn Sina) / Esomarfan (Marfan) / Esomenta (RAK) / Esomep (ACI) / Esomeprazol Genfar (Genfar S.A) / Esopral (Maquifarma) / Esorest (Centaur) / Inexium paranova / Lucen (Malesci) / Nexiam (AstraZeneca) |
| lansoprazole | Agopton (Takeda) / Bamalite / Lansoloc (Cipla Medpro) / Lanzol (Cipla) / Lanzopral (Pharma Investi) / Lanzopran (Ranbaxy) / Limpidex (Sigma-Tau) / Monolitum (Salvat) / Ogast (Takeda) / Ogastro (Abbott) / Opiren (Almirall) / Prevacid 24HR (Novartis) / Prosogan (Takeda) / Takepron (Takeda) / Ulpax (Hormona) / Zoprol (Toprak) / Zoton (Pfizer) |
| dexlansoprazole | Dexilant |
| rabeprazole | Pariet |
| pantoprazole | Pantozol |
| ilaprazole | Noltec |

**Table S3: The number and RORs of HARE reports of Omeprazole in different PT**

| **PT** | **a** | **ROR**  **(95% two-sided CI)** | **P** |
| --- | --- | --- | --- |
| Acute hepatic failure | 26 | 2.014 (1.370-2.962) | 0.001 |
| Alanine aminotransferase increased | 97 | 1.956 (1.601-2.388) | 0.000 |
| Aspartate aminotransferase increased^a^ | 53 | 1.279 (0.976-1.675) | 0.082 |
| Bilirubin conjugated increased | 7 | 3.406 (1.618-7.171) | 0.005 |
| Blood bilirubin increased | 43 | 1.706 (1.264-2.302) | 0.001 |
| Cholestasis | 80 | 5.131 (4.114-6.401) | 0.000 |
| Hepatic encephalopathy^a^ | 16 | 1.583 (0.968-2.587) | 0.080 |
| Hepatic enzyme increased^a^ | 60 | 1.155 (0.896-1.489) | 0.265 |
| Hepatic failure | 40 | 1.396 (1.023-1.904) | 0.035 |
| Hepatic function abnormal | 47 | 1.484 (1.114-1.977) | 0.007 |
| Hepatic necrosis | 15 | 5.515 (3.311-9.183) | 0.000 |
| Hepatitis | 42 | 1.992 (1.471-2.698) | 0.000 |
| Hepatitis acute | 25 | 4.346 (2.929-6.448) | 0.000 |
| Hepatitis cholestatic | 27 | 5.902 (4.034-8.634) | 0.000 |
| Hepatitis fulminant | 8 | 3.254 (1.622-6.528) | 0.004 |
| Hepatitis toxic | 9 | 3.821 (1.981-7.370) | 0.001 |
| Hepatotoxicity | 51 | 2.789 (2.117-3.674) | 0.000 |
| Hyperbilirubinaemia^a^ | 7 | 0.693 (0.330-1.454) | 0.429 |
| Jaundice | 63 | 2.319 (1.810-2.972) | 0.000 |
| Jaundice cholestatic | 13 | 4.029 (2.332-6.690) | 0.000 |
| Liver function test abnormal | 104 | 3.588 (2.957-4.354) | 0.000 |
| Liver injury | 25 | 1.672 (1.128-2.477) | 0.019 |
| Transaminases abnormal | 3 | 4.278 (1.371-13.351) | 0.035 |
| Transaminases increased | 52 | 2.299 (1.750-3.021) | 0.000 |

HRAE, Hepatotoxicity-Related Adverse Effect; PT, Preferred Terms; a, not defined a signal

**Table S4: The number and RORs of HARE reports of Esomeprazole in different PT**

| **PT** | **a** | **ROR**  **(95% two-sided CI)** | **P** |
| --- | --- | --- | --- |
| Acute hepatic failure | 15 | 4.026 (2.424-6.687) | 0.000 |
| Alanine aminotransferase increased | 24 | 1.673 (1.120-2.499) | 0.017 |
| Aspartate aminotransferase increased | 24 | 2.006 (1.343-2.997) | 0.002 |
| Blood bilirubin increased^a^ | 11 | 1.510 (0.835-2.729) | 0.188 |
| Cholestasis | 96 | 21.556 (17.592-26.413) | 0.000 |
| Hepatic encephalopathy^a^ | 3 | 1.027 (0.331-3.186) | 0.770 |
| Hepatic enzyme increased | 26 | 1.735 (1.180-2.550) | 0.009 |
| Hepatic failure^a^ | 14 | 1.691 (1.001-2.858) | 0.054 |
| Hepatic function abnormal | 22 | 2.407 (1.583-3.660) | 0.000 |
| Hepatitis | 40 | 6.594 (4.829-9.006) | 0.000 |
| Hepatitis acute | 17 | 10.243 (6.352-16.519) | 0.000 |
| Hepatitis cholestatic | 27 | 20.487 (13.996-29.987) | 0.000 |
| Hepatitis fulminant | 4 | 5.632 (2.109-15.044) | 0.006 |
| Hepatotoxicity^a^ | 6 | 1.134 (0.509-2.526) | 0.661 |
| Hyperbilirubinaemia | 9 | 3.085 (1.603-5.936) | 0.003 |
| Jaundice | 31 | 3.957 (2.779-5.635) | 0.000 |
| Liver function test abnormal^a^ | 6 | 0.714 (0.320-1.589) | 0.601 |
| Liver injury | 37 | 8.602 (6.220-11.896) | 0.000 |
| Transaminases increased | 22 | 3.370 (2.213-5.124) | 0.000 |

HRAE, Hepatotoxicity-Related Adverse Effect; PT, Preferred Terms; a, not defined a signal

**Table S5: The number and RORs of HARE reports of Lansoprazole in different PT**

| **PT** | **a** | **ROR**  **(95% two-sided CI)** | **P** |
| --- | --- | --- | --- |
| Acute hepatic failure | 16 | 3.115 (1.906-5.091) | 0.000 |
| Alanine aminotransferase increased | 44 | 2.229 (1.657-2.999) | 0.000 |
| Aspartate aminotransferase increased^a^ | 23 | 1.394 (0.925-2.099) | 0.104 |
| Blood bilirubin increased^a^ | 13 | 1.295 (0.751-2.231) | 0.340 |
| Cholestasis | 92 | 13.387 (10.885-16.464) | 0.000 |
| Coma hepatic | 3 | 10.424 (3.340-32.532) | 0.003 |
| Hepatic encephalopathy | 15 | 3.730 (2.246-6.197) | 0.000 |
| Hepatic enzyme increased^a^ | 25 | 1.209 (0.816-1.791) | 0.000 |
| Hepatic failure | 28 | 2.457 (1.695-3.562) | 0.000 |
| Hepatic function abnormal | 68 | 5.423 (4.269-6.889) | 0.000 |
| Hepatitis | 77 | 9.236 (7.372-11.571) | 0.000 |
| Hepatitis acute | 30 | 13.129 (9.151-18.836) | 0.000 |
| Hepatitis cholestatic | 28 | 15.403 (10.596-22.389) | 0.000 |
| Hepatotoxicity^a^ | 5 | 0.686 (0.285-1.648) | 0.574 |
| Hyperbilirubinaemia | 20 | 4.979 (3.207-7.731) | 0.000 |
| Jaundice | 50 | 4.636 (3.509-6.126) | 0.000 |
| Jaundice cholestatic | 7 | 5.450 (2.592-11.460) | 0.000 |
| Liver function test abnormal | 29 | 2.509 (1.742-3.615) | 0.000 |
| Liver injury | 24 | 4.037 (2.702-6.032) | 0.000 |
| Transaminases increased^a^ | 9 | 0.998 (0.519-1.920) | 1.000 |

HRAE, Hepatotoxicity-Related Adverse Effect; PT, Preferred Terms; a, not defined a signal

**Table S6: The number and RORs of HARE reports of Rabeprazole in different PT**

| **PT** | **a** | **ROR**  **(95% two-sided CI)** | **P** |
| --- | --- | --- | --- |
| Acute hepatic failure | 5 | 4.775 (1.984-11.490) | 0.004 |
| Alanine aminotransferase increased | 14 | 3.486 (2.060-5.899) | 0.000 |
| Aspartate aminotransferase increased | 14 | 4.179 (2.470-7.073) | 0.000 |
| Cholestasis | 22 | 17.519 (11.497-26.696) | 0.000 |
| Hepatic encephalopathy | 4 | 4.877 (1.828-13.016) | 0.010 |
| Hepatic enzyme increased^a^ | 6 | 1.423 (0.638-3.171) | 0.327 |
| Hepatic failure^a^ | 4 | 1.718 (0.644-4.584) | 0.304 |
| Hepatic function abnormal | 24 | 9.432 (6.303-14.116) | 0.000 |
| Hepatitis | 5 | 2.921 (1.214-7.029) | 0.031 |
| Hepatitis fulminant | 8 | 40.240 (20.032-80.834) | 0.000 |
| Jaundice | 10 | 4.543 (2.440-8.461) | 0.000 |
| Liver function test abnormal^a^ | 5 | 2.119 (0.881-5.098) | 0.091 |
| Liver injury | 6 | 4.949 (2.219-11.035) | 0.002 |
| Transaminases increased | 6 | 3.268 (1.466-7.287) | 0.011 |

HRAE, Hepatotoxicity-Related Adverse Effect; PT, Preferred Terms; a, not defined a signal

**Table S7: The number and RORs of HARE reports of Pantoprazole in different PT**

| **PT** | **a** | **ROR**  **(95% two-sided CI)** | **P** |
| --- | --- | --- | --- |
| Acute hepatic failure | 41 | 4.684 (3.443-6.373) | 0.000 |
| Alanine aminotransferase increased | 70 | 2.079 (1.643-2.630) | 0.000 |
| Aspartate aminotransferase increased | 57 | 2.028 (1.563-2.631) | 0.000 |
| Bilirubin conjugated increased | 10 | 7.169 (3.841-13.380) | 0.000 |
| Blood bilirubin increased | 43 | 2.514 (1.863-3.394) | 0.000 |
| Cholestasis | 201 | 19.173 (16.642-22.089) | 0.000 |
| Hepatic encephalopathy | 28 | 4.083 (2.815-5.924) | 0.000 |
| Hepatic enzyme abnormal | 10 | 2.872 (1.543-5.348) | 0.003 |
| Hepatic enzyme increased | 120 | 3.420 (2.856-4.096) | 0.000 |
| Hepatic failure | 44 | 2.263 (1.682-3.044) | 0.000 |
| Hepatic function abnormal^a^ | 26 | 1.208 (0.822-1.776) | 0.332 |
| Hepatic necrosis | 17 | 9.208 (5.700-14.875) | 0.000 |
| Hepatitis | 72 | 5.043 (3.997-6.364) | 0.000 |
| Hepatitis acute | 23 | 5.890 (3.905-8.886) | 0.000 |
| Hepatitis cholestatic | 70 | 22.611 (17.794-28.733) | 0.000 |
| Hepatitis fulminant | 29 | 17.399 (12.017-25.191) | 0.000 |
| Hepatitis toxic | 7 | 4.377 (2.080-9.210) | 0.001 |
| Hepatotoxicity | 37 | 2.980 (2.156-4.118) | 0.000 |
| Hyperammonaemia | 19 | 5.695 (3.623-8.9520 | 0.000 |
| Hyperbilirubinaemia^a^ | 6 | 0.874 (0.392-1.947) | 1.000 |
| Jaundice | 64 | 3.475 (2.716-4.445) | 0.000 |
| Jaundice cholestatic | 10 | 4.564 (2.449-8.507) | 0.000 |
| Liver function test abnormal^a^ | 19 | 0.962 (0.613-1.509) | 0.985 |
| Liver injury | 30 | 2.957 (2.064-4.234) | 0.000 |
| Transaminases increased | 81 | 5.290 (4.248-6.588) | 0.000 |

HRAE, Hepatotoxicity-Related Adverse Effect; PT, Preferred Terms; a, not defined a signal

**Table S8: Ranking of indications and concomitant medications in Pantoprazole reports about hepatic encephalopathy**

| **Indication** | **Number of occurrences (n)** | **Rank** | **concomitant medications** | **Number of occurrences (n)** |
| --- | --- | --- | --- | --- |
| UNKNOWN | 378 | **1** | CUBICIN | 23 |
| SKIN INFECTION | 45 | **2** | ULTRAVIST | 22 |
| CHENST SCAN | 19 | **3** | VANCOMYCIN | 22 |
| GASTROOESOPHAGEAL REFLUX DISEASE | 6 | **4** | QUESTRAN | 20 |
| COMPUTERISED TOMOGRAM THORAX | 2 | **5** | RACECADOTRIL | 19 |
| HEPATIC CIRRHOSIS | 2 | **6** | NEFOPAM | 19 |
| HYPERTENSION | 2 | **7** | LINEZOLID | 19 |
| SLEEP DISORDER | 1 | **8** | PRIMPERAN | 17 |
| HEPATITIS B VIRUS TEST | 1 | **9** | PIPERACILLIN/TAZOBACTAM | 17 |
|  |  | **10** | RIFADIN | 15 |
|  |  | **11** | METOCLOPRAMIDE | 10 |
|  |  | **12** | SIMBICORT TURBUHALER | 9 |
|  |  | **13** | RIFAMPICIN | 7 |
|  |  | **14** | DAPTOMYCIN | 6 |
|  |  | **15** | SYMBICORT | 6 |
|  |  | **16** | PREVACID | 5 |
|  |  | **17** | PARACETAMOL/TRAMADOL | 4 |
|  |  | **18** | COLESTYRAMINE | 4 |
|  |  | **19** | DIMETINDENE | 3 |
|  |  | **20** | BUDESONIDE | 3 |

**Table S9: Ranking of indications (Top 10) in Pantoprazole reports about major PT**

| **PT1: BILIRUBIN CONJUGATED INCREASED** | | **PT2: CHOLESTASIS** | | **PT3: HEPATIC NECROSIS** | | **PT4: HEPATITIS ACUTE** | |
| --- | --- | --- | --- | --- | --- | --- | --- |
| **Indication** | **Number of occurrences (n)** | **Indication** | **Number of occurrences (n)** | **Indication** | **Number of occurrences (n)** | **Indication** | **Number of occurrences (n)** |
| PRODUCT USED FOR UNKNOWN INDICATION | 14 | PRODUCT USED FOR UNKNOWN INDICATION | 385 | PRODUCT USED FOR UNKNOWN INDICATION | 47 | PRODUCT USED FOR UNKNOWN INDICATION | 13 |
| PAIN | 12 | HYPERTENSION | 102 | HIV INFECTION | 26 | ACUTE CORONARY SYNDROME | 11 |
| ACUTE MYELOID LEUKAEMIA | 6 | PROPHYLAXIS | 86 | PROPHYLAXIS | 13 | GASTROOESOPHAGEAL REFLUX DISEASE | 8 |
| INSOMNIA | 6 | PAIN | 56 | POSTOPERATIVE CARE | 12 | CORONARY ARTERY DISEASE | 8 |
| LUNG CANCER METASTATIC | 5 | ANXIETY | 45 | ATRIAL FIBRILLATION | 12 | THROMBOSIS PROPHYLAXIS | 5 |
| PROPHYLAXIS AGAINST GASTROINTESTINAL ULCER | 5 | INFECTION | 37 | GASTROOESOPHAGEAL REFLUX DISEASE | 7 | URINARY TRACT INFECTION | 5 |
| PROPHYLAXIS | 4 | THROMBOSIS PROPHYLAXIS | 27 | OESOPHAGEAL CANDIDIASIS | 6 | ULCER | 5 |
| CANDIDA INFECTION | 4 | GASTROOESOPHAGEAL REFLUX DISEASE | 27 | HYPERTENSION | 6 | PROPHYLAXIS AGAINST GASTROINTESTINAL ULCER | 5 |
| HYPERTENSION | 2 | HIV INFECTION | 26 | URINARY TRACT INFECTION | 6 | PAIN | 4 |
| INTESTINAL OBSTRUCTION | 2 | PROPHYLAXIS AGAINST GASTROINTESTINAL ULCER | 24 | ULCER | 5 | COLORECTAL CANCER | 3 |

PT, Preferred Terms

**Continue:**

| **PT5:** **HEPATITIS CHOLESTATIC** | | **PT6:** **HEPATITIS FULMINANT** | | **PT7:** **HEPATITIS** | | **PT8:** **TRANSAMINASES INCREASED** | |
| --- | --- | --- | --- | --- | --- | --- | --- |
| **Indication** | **Number of occurrences (n)** | **Indication** | **Number of occurrences (n)** | **Indication** | **Number of occurrences (n)** | **Indication** | **Number of occurrences (n)** |
| PRODUCT USED FOR UNKNOWN INDICATION | 162 | PRODUCT USED FOR UNKNOWN INDICATION | 106 | PRODUCT USED FOR UNKNOWN INDICATION | 127 | PRODUCT USED FOR UNKNOWN INDICATION | 348 |
| PROPHYLAXIS | 33 | HIV INFECTION | 26 | PAIN | 27 | PAIN | 23 |
| MYOCARDIAL INFARCTION | 33 | PAIN | 21 | HIV INFECTION | 26 | POST HERPETIC NEURALGIA | 20 |
| HIV INFECTION | 26 | PROPHYLAXIS | 15 | PROPHYLAXIS | 25 | HYPERTENSION | 17 |
| SEPSIS | 25 | ATRIAL FIBRILLATION | 12 | SEDATION | 24 | MUSCLE RIGIDITY | 15 |
| ARRHYTHMIA | 14 | GASTROOESOPHAGEAL REFLUX DISEASE | 7 | PROPHYLAXIS AGAINST GASTROINTESTINAL ULCER | 23 | INFECTION | 14 |
| OESOPHAGITIS | 13 | OESOPHAGEAL CANDIDIASIS | 6 | CARDIAC FAILURE | 21 | MENTAL DISORDER | 11 |
| ATRIAL FIBRILLATION | 13 | PNEUMONIA ASPIRATION | 6 | MENTAL DISORDER | 18 | CHRONIC OBSTRUCTIVE PULMONARY DISEASE | 10 |
| HYPERTENSION | 11 | HYPERTENSION | 6 | HYPERTENSION | 17 | GASTRITIS | 9 |
| THROMBOSIS PROPHYLAXIS | 10 | GASTRITIS | 5 | CHRONIC OBSTRUCTIVE PULMONARY DISEASE | 16 | ACUTE MYELOID LEUKAEMIA | 9 |

PT, Preferred Terms
